# PCR-RFLP assay as an option for primary HPV test

**DOI:** 10.1590/1414-431X20177098

**Published:** 2018-03-26

**Authors:** L. Golfetto, E.V. Alves, T.R. Martins, T.C.M. Sincero, J.B.S. Castro, C. Dannebrock, J.G. Oliveira, J.E. Levi, A.S.C. Onofre, M.L. Bazzo

**Affiliations:** 1 Laboratório de Biologia Molecular, Microbiologia e Sorologia, Universidade Federal de Santa Catarina, Florianópolis, SC, Brasil; 2 Laboratório de Virologia, Instituto de Medicina Tropical, Universidade de São Paulo, São Paulo, SP, Brasil; 3 Departamento de Análises Clínicas, Universidade Federal de Santa Catarina, Florianópolis, SC, Brasil; 4 Posto Central, Secretaria Municipal de Saúde, São Miguel do Oeste, SC, Brasil; 5 Laboratório Prevent Citopatologia, São Miguel do Oeste, SC, Brasil; 6 Grupo de Pesquisa em Imunologia Celular e Molecular, Instituto René Rachou, Fundação Oswaldo Cruz, Belo Horizonte, MG, Brasil

**Keywords:** Cervical cancer, HPV, Screening, PCR-RFLP, PapilloCheck^®^ microarray

## Abstract

Persistent human papillomavirus (HPV) infection is an essential factor of cervical cancer. This study evaluated the analytical performance of restriction fragment length polymorphism polymerase chain reaction (PCR-RFLP) assay compared to PapilloCheck^®^ microarray to identify human papilloma virus (HPV) in cervical cells. Three hundred and twenty-five women were analyzed. One sample was used for conventional cytology and another sample was collected using BD SurePath™ kit for HPV tests. Eighty samples (24.6%) were positive for HPV gene by PCR-Multiplex and were then submitted to PCR-RFLP and PapilloCheck^®^ microarray. There was a genotyping agreement in 71.25% (57/80) on at least one HPV type between PCR-RFLP and PapilloCheck^®^ microarray. In 22 samples (27.5%), the results were discordant and those samples were additionally analyzed by DNA sequencing. HPV 16 was the most prevalent HPV type found in both methods, followed by HPVs 53, 68, 18, 39, and 66 using PCR-RFLP analysis, and HPVs 39, 53, 68, 56, 31, and 66 using PapilloCheck^®^ microarray. In the present study, a perfect agreement using Cohen's kappa (*κ*) was found in HPV 33 and 58 (*κ*=1), very good for HPV 51, and good for types 16, 18, 53, 59, 66, 68, 70, and 73. PCR-RFLP analysis identified only 25% (20/80) HPV coinfection, and PapilloCheck^®^ microarray found 62.5% (50/80). Our Cohen's kappa results indicate that our in-house HPV genotyping testing (PCR-RFLP analysis) could be applied as a primary HPV test screening, especially in low income countries. If multiple HPV types are found in this primary test, a more descriptive test, such as PapilloCheck^®^ microarray, could be performed.

## Introduction

Cervical cancer was responsible for an estimated 530,000 diagnoses and 266,000 deaths in 2012, the most common type of gynecological tumor worldwide (1). In Brazil, cervical cancer is the third most common tumor in women and the fourth cause of death. In 2013, 5,430 women died from cervical cancer, and in 2016 16,340 new cases are estimated in Brazil (2). Persistent human papillomavirus (HPV) infection is an essential factor of cervical cancer (3). Two hundred different types of HPV have been discovered (4). From those, about 51 types are considered either high-risk (HR) or low-risk (LR) genital HPVs types associated with benign, precancerous or cancer lesions (5). This discovery is changing the target for reducing the high mortality of cervical cancer, such as the introduction of the HPV vaccine and/or application of the HPV test as a primary screening test.

The introduction of HPV vaccine is stimulating developed countries to change their cervical screening program from cytology to HPV testing as a primary screening test (6–10). However, various strategies have been proposed to achieve better performance in the detection of precancerous lesions and optimize balance between benefits and harms (11–13). Some countries are presently discussing which test should be used to improve sensitivity and specificity for cervical screening program.

PapilloCheck^®^ microarray and restriction fragment length polymorphism polymerase chain reaction (PCR-RFLP) assay are HPV tests based on different methodology that could be applied in screening programs. PapilloCheck^®^ microarray is based on PCR amplification of a fragment of approximately 350pb from the E1 region of HPV genomes. The kit simultaneously detects 24 different HPVs (6, 11, 16, 18, 31, 33, 35, 39, 40, 42, 43, 44/55, 45, 51, 52, 53, 56, 58, 59, 66, 68, 70, 73, and 82) (14). PCR-RFLP assay is based on the amplification of a fragment of L1 gene using PGMY09/11 primers and subsequent RFLP analysis of four restriction enzymes (*Pst*I, *Hae*III, *Dde*I, and *Rsa*I). The two methods are different in sensitivity, specificity, and costs. This study evaluated the analytical performance of PCR-RFLP assay compared to the PapilloCheck^®^ microarray for HPV identification.

## Material and Methods

### Study population

From November 2011 to March 2013, 990 women were attended for cervical screening in public clinics in São Miguel do Oeste, SC, Brazil. Three hundred and twenty-five of those women accepted to participate in our study. Two samples were collected from each woman. One sample was used for conventional cytology for routine diagnosis. Another sample was collected with BD SurePath™ kit for HPV tests. The routine cytological analysis was performed by Papanicolaou staining, analyzed and classified according to the 2001 Bethesda system: NILM (negative for intraepithelial lesion or malignancy), ASC-US (atypical squamous cells of undetermined significance), ASC-H (atypical squamous cells – cannot exclude HSIL), LSIL (low-grade squamous intraepithelial lesion), HSIL (high-grade squamous intraepithelial lesion), and ICC (invasive cervical cancer) (15). The absence of biopsies are a limitation of the present study. The liquid based sample was used for an in-house HPV genotyping testing (PCR-RFLP analysis) and PapilloCheck^®^ microarray. For discrepancies of HPV results, DNA sequencing of L1 amplified fragments was performed.

### DNA extraction

To select which method should be applied for DNA extraction, we used QiaAmp^®^ DNA mini Kit (Qiagen, Germany) (16), phenol–chloroform extraction, guanidinium thiocyanate extraction (17), and ammonium acetate extraction (18). The best results were obtained by the ammonium acetate method.

DNA was extracted from cervical cytology vials (SurePath™, USA). A 1-mL aliquot was pelleted by centrifugation at 12,000 *g* for 5 min at 25°C. The preservative fluid was removed and cells were resuspended in 180 μL sterile phosphate buffered saline (PBS). Subsequently, 20 µL of Proteinase K (Qiagen) and 180 µL of AL buffer (Qiagen) were added to these cells, vortexed and heated at 56°C under agitation (1200 rpm) for 1 h and at 90°C for another hour. Two hundred microliters of 2 M ammonium acetate (Sigma, USA) were added to the cell lysate, submitted to an ice bath for 5 min, and centrifuged at 12,000 *g* for 4 min at 25°C. Then, the supernatant was transferred to another tube and 600 µL of isopropanol (Sigma) was added. The cells lysate was homogenized for 20 inversions and centrifuged at 12,000 *g* for 4 min at 25°C. The supernatant was discarded; the pellet was washed with 1 mL of 70% ethanol (Sigma) and centrifuged at 12,000 *g* for 4 min at 25°C. The supernatant was removed and the pellet was maintained at 60°C until complete evaporation of the ethanol. DNA was suspended in 50 µL of elution buffer Tris-EDTA and stored at –20°C until further use. DNA quality control was determined by NanoVue spectrophotometry (GE Healthcare, United Kingdom).

### Polymerase chain reaction (PCR)

Multiplex PCR with PGMY09/11 and PCO4/GH20 primers (19) was performed on a final reaction volume of 25 µL. The reaction was carried out with 20 mM Tris-HCl, pH 8.4, 50 mM KCl, 5.7% glycerol, 0.04 mM of each PGMY09/11 primer (Life Tecnologies™, USA), 0.2 mM of each PCO4/GH20 primer (Life Tecnologies™), 2.5 mM of MgCl_2_, 200 μM of dNTP (Life Tecnologies™), 2U Taq Platinum DNA Polymerase (Life Tecnologies™, Brazil), and 5.0 μL of DNA. The target DNA was amplified by PCR (Mastercycle Personal^®^ Eppendorf) and reaction was carried out with a denaturation step at 95°C for 10 min, 40 cycles of 1 min at 95°C, 1 min at 55°C, 1 min at 72°C,and final extension at 72°C for 10 min. Plasmids containing HPV-33 L1 gene were used as positive control and DNase- and RNase-free water was used as negative control in all amplifications.

### Restriction fragment length polymorphism (RFLP)

HPV DNA positive samples were submitted to a PCR reaction with PGMY09/11 primers to perform PCR-RFLP for HPV genotyping. Reaction was performed according to Nobre et al. (20), with the enzymes *Pst*I (Promega, USA), *Hae*III (Promega, Madison USA), *Dde*I (Promega), and *Rsa*I (Promega).

### PapilloCheck^®^ microarray

All positive HPV PCR-Multiplex samples were submitted to PapilloCheck^®^ microarray (Greiner Bio-One, Germany) 5μL of DNA eluate was used in the PapilloCheck^®^ microarray for each reaction. Specimens containing the target DNA are hybridized to specific oligonucleotide probes immobilized on a DNA chip and detected by the binding of a Cy5-dUTP labeled oligonucleotide probe to the tag sequence. The DNA chip was scanned by the CheckScanner apparatus at 532 and 635 nm wave lengths. This test detects HPV genotypes 6, 11, 16, 18, 31, 33, 35, 39, 40, 42, 43, 44, 45, 51, 52, 53.56, 58, 59, 66, 68, 70, 73, and 82. In addition, human ADAT1 gene (adenosine deaminase, tRNA specific 1) was used as an internal control to assess the quality of the DNA

### DNA sequencing

DNA sequencing of PGMY09/11 PCR fragments was performed for samples with discordant results between the two genotyping methods. The amplicons were purified with the PureLink^®^ PCR Purification kit (Life Tecnologies™, Germany) or with QIAquick Gel Extraction kit (Qiagen), according to the manufacturers' instructions. Automated DNA sequencing was performed in an ABI 3730 Genetic Analyzer sequencer (Applied Biosystems, USA). The accuracy of the DNA sequencing was evaluated through the CAP3 program based on Phred quality score (21).

Sequences were aligned and compared to those available in the GenBank database using the software Chromas Lite 2.1 (Technelysium, Australia). HPV type was identified based on >90% sequence homology over 449–458 nucleotides.

### Statistical analysis

To determine the correlation between PCR-RFPL and PapilloCheck^®^ microarray, Kappa test was performed and the reference values adopted were determined as proposed by Altman (22). A Kappa value of 0 indicates no agreement and a value of 1 indicates perfect agreement. Values from 0.00–0.20 indicate poor agreement, 0.21–0.40 fair, 0.41–0.60 moderate, 0.61–0.80 good, and 0.81–0.99 very good agreement.

### Ethical approval

This cross-sectional descriptive study was approved by Research Ethical Committee of the Universidade Federal de Santa Catarina (process No. 2155), and participants provided written informed consent to the study protocol.

## Results

The cytological diagnosis from the 325 sexually active women (average age 37 years; range 14–79 years), included 313 (96.3%) women with NILM and 11 (3.4%) with some cytological abnormality as follows: 4 ASCUS, 4 LSIL, 3 ASC-H. All samples with abnormal cytology were HR-HPV positive (Table 1).

**Table 1.** List of HPV positive samples with cytological abnormalities.

Cytology	PCR-RFLP	PapilloCheck^®^ microarray
ASC-H	HPV 16, 33	HPV 16, 33, 39, 51, 52
ASC-H	HPV 58	HPV 58
ASC-H	HPV 16	HPV 16
ASC-US	HPV 35	HPV 35, 68
ASC-US	HPV 16, 69	HPV 16
ASC-US	Inconclusive	HPV 16, 56, 39, 82
ASC-US	HPV 53	HPV 53, 31
LSIL	HPV 16, 18	HPV 16, 18, 39
LSIL	HPV 53	HPV 53
LSIL	HPV 16	HPV 16, 35
LSIL	HPV 45, 66	HPV 45, 56, 66

PCR-RFLP: restriction fragment length polymorphism polymerase chain reaction; ASC-H: atypical squamous cells (cannot exclude high-grade squamous intraepithelial lesion); ASC-US: atypical squamous cells of undetermined significance; LSIL: low-grade squamous intraepithelial lesion

All 325 samples were positive for β-globin gene (control) and 80 samples (24.6%) were positive for HPV gene by PCR-Multiplex. Those 80 samples were then submitted to PCR-RFLP and PapilloCheck^®^ microarray. Of those, 22 (35.0%) were additionally analyzed by DNA sequencing.

### PCR-RFLP and PapilloCheck^®^ microarray

**Figure 1.**
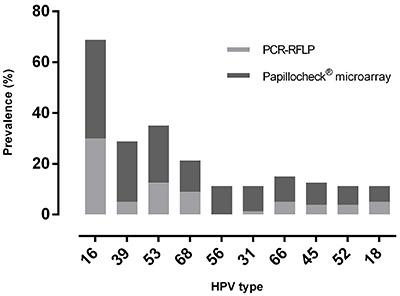
Prevalence of the most frequent high risk-HPV types by PapilloCheck^®^ microarray and restriction fragment length polymorphism polymerase chain reaction (PCR-RFLP).

From the 80 HPV positive samples, 70 (87.50%) were genotyped by PCR-RFLP and 10 (12.5%) were inconclusive. The PCR-RFLP method identified 28 different HPV types. From those, 17 were classified as HR-HPV (16, 18, 31, 33, 35, 39, 45, 51, 52, 53, 58, 59, 66, 68, 69, 70, and 73) and 11 were classified as LR-HPV (06, 11, 32, 44, 55, 61, 62, 74, 83, 84, and 89). In HR-HPV group (Figure 1), HPV 16 was the most prevalent type (30%), followed by types 53 (12.5%), 68 (8.8%), 18 (5%), 39 (5%), 66 (5%), and 45, 51, 52 (3.8% each). The prevalent types in LR-HPV group were 61 (3.8%), 62 (2.5%), 89 (2.5%), and 6, 11 (1.3% each). Single infection was observed in 75% of samples.

PapilloCheck^®^ microarray genotyped 72 (90.0%) samples, and 8 (10.0%) were not identified. The PapilloCheck*^®^* microarray identified all 24 HPV types described by the manufacturer. In HR-HPV group (Figure 1), HPV 16 was the most prevalent type (38.8%), followed by types 39 (23.8%), 53 (22.5%), 68 (12.5%), 56 (11.3%), 31 (10%), 66 (10%) 44/55 (8.8%), 43 (7.5%), 52 (7.5%), 18 (6.3%), and 6, 11, 35, 51, 73 (3.8% each). The prevalent types in LR-HPV group were 44/55 (3.8%), 43 (7.5%), 6 (5.0%), and 11 (5.0%). Multiple infections were observed in 62.5% of samples.

### Agreement between PCR-RFLP and Papillocheck^®^ microarray

There was genotyping agreement in 71.25% (57/80) on at least one HPV type between PCR-RFLP and PapilloCheck^®^ microarray. In 22 of the 80 samples (27.5%), the results were discordant. All discordant results were additionally analyzed by DNA sequencing. From those 22 samples, 10 were inconclusive by PCR-RFLP. Eight samples were not identified by PapilloCheck^®^ microarray. In one sample, HPV 68 was identified by PCR-RFLP, but HPV 39 by PapilloCheck^®^ microarray. Two samples were positive for HPV 62 by PCR-RFLP. HPV 53 was detected in one sample and HPV 39 and 43 in another sample by PapilloCheck^®^ microarray. HPV 61 was found in one sample by PCR-RFLP and HPV 53 and 44/55 by PapilloCheck^®^ microarray. HPV 32 was revealed by PCR-RFLP in one sample and HPV 56 in another sample by PapilloCheck^®^ microarray (Table 2). From the inconclusive results by PCR-RFLP, one was negative and nine were genotyped by PapilloCheck^®^ microarray. Multiple HPV infections were detected in 7 samples using PapilloCheck^®^ microarray.

**Table 2.** DNA sequencing results compared with discordant results of PCR-RFLP and PapilloCheck^®^ microarray.

PCR-RFLP	PapilloCheck^®^ microarray	DNA sequencing
HPV 61	Negative	HPV 61
HPV 84	Negative	HPV 84
Inconclusive	HPV 56	HPV 62
HPV 89	Negative	HPV 89
HPV 61	HPV 53, 44/55	HPV 61
HPV 61	Negative	HPV 61
Inconclusive	HPV 11, 35, 42, 73	HPV 35
HPV 89	Negative	HPV 89
HPV 62	HPV 53	HPV 62
HPV 62	HPV 39, 43	HPV 62
HPV 68	HPV 39	HPV 68
HPV 74	Negative	HPV 74
HPV 32	HPV 11 e 39	HPV 32
Inconclusive	HPV 16 e 66	HPV 61
Inconclusive	Negative	HPV 89
HPV 83	Negative	HPV 83
Inconclusive	HPV 06, 16, 43, 44/55, 53, 56	HPV 06
Inconclusive	HPV 16, 39	HPV 16
Inconclusive	HPV 16, 39	HPV 16
Inconclusive	HPV 43, 45, 59, 68	HPV 61
Inconclusive	HPV 56, 73, 66	HPV 66
Inconclusive	HPV 56	HPV 62

PCR-RFLP: restriction fragment length polymorphism polymerase chain reaction.

Samples in which HPV types were not identified by PapilloCheck^®^ microarray, genotyping was performed by PCR-RFLP and/or DNA sequencing: HPV 89 (three samples), HPV 61 (two samples), and HPVs 74, 83, 84 (one sample each). All HPV types revealed by PCR-RFLP were in concordance to DNA sequencing (Table 2).

Kappa test was applied to analyze the agreement level between PCR-RFLP and PapilloCheck^®^ microarray. The genotyping agreement between the methods was considered perfect for HPV types 33 and 58; very good for type 51; good for types 16, 18, 53, 59, 66, 68, 70, and 73; moderate for types 45 and 52, fair for types 6, 11, 35, and 44/55; and poor for types 31 and 39. It was not possible to determine agreement for HPV types 40, 42, 43, 56, and 82 because these were identified only by PapilloCheck^®^ microarray (Table 3).

**Table 3.** Degree of agreement between PCR-RFLP and PapilloCheck^®^ microarray.

HPV types	Kappa	P* (95%CI)	Agreement	HPV classification
HPV 33	1.000	0.025 (0.000-0.059)	Perfect	HR
HPV 58	1.000	<0.001 (0.000-0.059)	Perfect	HR
HPV 51	0.851	<0.001 (0.000-0.037)	Very good	HR
HPV 18	0.787	<0.001 (0.000-0.037)	Good	HR
HPV 16	0.668	<0.001 (0.000-0.037)	Good	HR
HPV 66	0.643	<0.001 (0.000-0.037)	Good	HR
HPV 59	0.661	0.013 (0.000-0.037)	Good	HR
HPV 70	0.661	0.038 (0.000-0.079)	Good	HR
HPV 53	0.660	<0.001 (0.000-0.037)	Moderate	HR
HPV 73	0.655	0.013 (0.000-0.037)	Moderate	HR
HPV 45	0.578	<0.001 (0.000-0.037)	Moderate	HR
HPV 68	0.541	<0.001 (0.000-0.037)	Moderate	HR
HPV 52	0.415	<0.001 (0.000-0.037)	Moderate	HR
HPV 06	0.388	0.013 (0.00-0.037)	Fair	LR
HPV 11	0.274	0.100 (0.034-0.166)	Fair	LR
HPV 35	0.274	0.05 (0.002-0.098)	Fair	HR
HPV 44/55	0.233	0.100 (0.034-0.166)	Fair	LR
HPV 31	0.205	0.163 (0.082-0.243)	Poor	HR
HPV 39	0.194	0.025 (0.000-0.059)	Poor	HR

* P values for Kappa test. PCR-RFLP: restriction fragment length polymorphism polymerase chain reaction; HR: high risk; LR: low risk.

## Discussion

The present study found 24.6% (80/325) positivity for HPV gene by PCR-Multiplex. Compared with other Brazilian reports that applied PCR-RFLP, discrepancies between regions were present. Miranda et al. (24) found 11% positivity for HPV in Ouro Preto, Coser et al. (25) 15.7% in Rio Grande do Sul, while Fernandes et al. (26) 48% in Rio Grande do Norte. To improve our results, we compared PCR-RFLP and Papillocheck^®^ microarray. Thirty-five percent (22/80) of those positive samples revealed discrepancies between PCR-RFLP and PapilloCheck^®^ microarray.

HPV 16 was the most prevalent HPV type found in both methods applied in this study, followed by HPVs 53, 68, 18, 39, and 66 using PCR-RFLP analysis and HPVs 39, 53, 68, 56, 31, and 66 using PapilloCheck^®^ microarray. HPV 16 was the most prevalent HPV type found in Brazilian studies that applied PCR-RFLP analysis (24–26) as well as PapilloCheck^®^ microarray assay (27,28). Several global and national studies reveal this highest prevalence for HPV 16 in women with normal and with abnormal cytology (27,29–32).

In this study, HPV 53 was found in 12.5% by PCR-RFLP analysis, while in 22.5% of samples by PapilloCheck^®^ microarray. Using PapilloCheck^®^ microarray, HPV 39 was revealed in 23.8%, but only in 5% of samples by PCR-RFLP analysis. Other types of HPVs, such as 68 and 18, did not show relevant differences in their prevalence. Martins et al. (27) reported 19.1 and 28.8% prevalence of HPV 56 among HPV+ women bearing NILM and LSIL cytology, respectively.

Some studies have reported that HPV 56 was the second most frequent type (27,33,34) while others revealed low frequency, which corroborate to the current study (35,36). This discrepancy could be attributed to a higher sensitivity of PapilloCheck^®^ microarray compared to PCR-RFLP analysis or due to its reduced specificity (37).

DNA sequencing was applied for discordant results from PCR-RFLP and PapilloCheck^®^ microarray. In one case, HPV 68 was found by PCR-RFLP and DNA sequencing, while HPV 39 was found only by PapilloCheck^®^ microarray. Those HPV types belong to same family, *alphapapillomavirus*, genus α7 in the phylogenetic tree (23,38), which could justify the different results. To confirm the DNA sequencing, cloning was performed using 450 bp fragment amplified by primers and subsequently sequenced using PGMY09/11, which revealed HPV types 61 (5 cases), HPV 62 (4 cases), and HPV 32 (1 case) in the clones.

In the present study, a perfect agreement was found for HPV 33 and 58 (*κ*=1), very good for HPV 51, and good for types 16, 18, 53, 59, 66, 68, 70, and 73. PCR-RFLP analysis identified only 25% (20/80) HPV coinfection, and PapilloCheck^®^ microarray found 62.5% (50/80). Results indicated that our in-house HPV genotyping testing (PCR-RFLP analysis) could be applied as a primary HPV test screening, especially in low income countries. Probably, the small number of patients limited our results. However, if multiple HPV types are found in this primary test, a more descriptive test, such as PapilloCheck^®^ microarray, could be performed. Currently, there is no gold standard for HPV typing (39,40) and the method should be chosen for clinical purpose based on its advantages and disadvantages.
